# Spinal Palpation Error and Its Impact on Skin Marker-Based Spinal Alignment Measurement in Adult Spinal Deformity

**DOI:** 10.3389/fbioe.2021.687323

**Published:** 2021-06-23

**Authors:** Pieter Severijns, Thomas Overbergh, Stefan Schmid, Lieven Moke, Lennart Scheys

**Affiliations:** ^1^Department of Development and Regeneration, Faculty of Medicine, Institute for Orthopaedic Research and Training, KU Leuven, Leuven, Belgium; ^2^Department of Rehabilitation Sciences, KU Leuven, Leuven, Belgium; ^3^Spinal Movement Biomechanics Group, Division of Physiotherapy, Department of Health Professions, Bern University of Applied Sciences, Bern, Switzerland; ^4^Division of Orthopaedics, University Hospitals Leuven, Leuven, Belgium

**Keywords:** spinal palpation error, adult spinal deformity, marker-based spinal alignment measurement, marker placement, thoracolumbar, lumbar, spinal level identification, motion analysis

## Abstract

Spinal alignment measurement in spinal deformity research has recently shifted from using mainly two-dimensional static radiography toward skin marker-based motion capture approaches, allowing three-dimensional (3D) assessments during dynamic conditions. The validity and accuracy of such skin marker-based methods is highly depending on correct marker placement. In this study we quantified, for the first time, the 3D spinal palpation error in adult spinal deformity (ASD) and compared it to the error in healthy spines. Secondly, the impact of incorrect marker placement on the accuracy of marker-based spinal alignment measurement was investigated. 3D, mediolateral and inferosuperior palpation errors for thoracolumbar and lumbar vertebral levels were measured on biplanar images by extracting 3D positions of skin-mounted markers and their corresponding anatomical landmarks in 20 ASD and 10 healthy control subjects. Relationships were investigated between palpation error and radiographic spinal alignment (lordosis and scoliosis), as well as body morphology [BMI and soft tissue (ST) thickness]. Marker-based spinal alignment was measured using a previously validated method, in which a polynomial is fit through the marker positions of a motion trial and which allows for radiograph-based marker position correction. To assess the impact of palpation error on spinal alignment measurement, the agreement was investigated between lordosis and scoliosis measured by a polynomial fit through, respectively, (1) the uncorrected marker positions, (2) the palpation error-corrected (optimal) marker positions, and (3) the anatomically corrected marker positions (toward the vertebral body), and their radiographic equivalents expressed as Cobb angles (ground truth), using Spearman correlations and root mean square errors (RMSE). The results of this study showed that, although overall accuracy of spinal level identification was similar across groups, mediolateral palpation was less accurate in the ASD group (ASD_mean_: 6.8 mm; Control_mean_: 2.5 mm; *p* = 0.002). Significant correlations with palpation error indicated that determining factors for marker misplacement were spinal malalignment, in particular scoliotic deformity (*r* = 0.77; *p* < 0.001), in the ASD group and body morphology [i.e., increased BMI (*r*_s_ = 0.78; *p* = 0.008) and ST thickness (*r*_s_ = 0.66; *p* = 0.038)] in healthy spines. Improved spinal alignment measurements after palpation error correction, shows the need for radiograph-based marker correction methods, and therefore, should be considered when interpreting spinal kinematics.

## Introduction

Spinal alignment measurement in spinal deformity research has recently shifted from using mainly two-dimensional (2D) static radiography (Schwab et al., 2012; Smith et al., 2013; Ailon et al., 2015) toward skin marker-based motion capture approaches. This allows three-dimensional (3D) assessment during both static positions and dynamic conditions, including daily life motor tasks (Schmid et al., 2016; Diebo et al., 2018; Severijns et al., 2020, 2021). However, the validity and accuracy of such skin marker-based methods is highly dependent on correct marker placement, which is known to be one of the main sources of variability in kinematic results (Della Croce et al., 2005; Gorton et al., 2009; McFadden et al., 2020). Nevertheless, information on spinal marker placement accuracy (i.e., palpation error) and its possible effect on spinal alignment measurements, in both healthy and deformed spines, is scarce.

Schmid et al. (2015) previously investigated the validity of skin marker-based spinal alignment measurement in adolescent idiopathic scoliosis (AIS) and observed systematic underestimations of the coronal curves. In addition, inaccurate marker placement was found to lead to an underestimation of spinous process-derived thoracolumbar and lumbar curves. Mean 2D palpation error over the entire spine in the inferosuperior and mediolateral direction was 8.2 mm and 1.3 mm, respectively (Schmid et al., 2015). Additionally, Severijns et al. (2020) recently introduced a method to quantify subject-specific spinal alignment in adult spinal deformity (ASD) allowing correction of the skin marker positions toward the positions of the corresponding vertebral bodies. They reported an underestimation of both sagittal and coronal curves when uncorrected skin marker positions were used. However, the impact of correcting the marker positions to their theoretical optimal skin position was not investigated (Severijns et al., 2020).

Data on the accuracy of identifying spinal structures (e.g., spinous processes) through manual palpation is also of importance in the treatment of spinal disorders, for instance to identify symptomatic levels, to assess intervertebral motion or to identify injection locations (Simmonds and Kumar, 1993; Broadbent et al., 2000; Nyberg and Russell Smith, 2013). However, even in non-deformed spines, results on the accuracy and reliability of these palpations are rather inconsistent, possibly due to differences in assessment methods (Haneline and Young, 2009; Kilby et al., 2012). Correct level identifications reported in the literature, varied from 29 to 71% and for mean palpation error values have been reported varying from 2.7 to 19.3 mm (Downey et al., 1999; Broadbent et al., 2000; Harlick et al., 2007; Kilby et al., 2012; Cooper et al., 2013). All these studies report 2D instead of 3D errors and, to the author’s knowledge, the palpation error in deformed adult spines specifically has not yet been investigated.

This study therefore aimed at quantifying the 3D spinal palpation error in deformed adult spines and to compare it to the error in healthy non-deformed spines. Moreover, we sought to explore underlying reasons for palpation error by investigating associations with radiographic alignment and body morphology parameters, i.e., the body mass index (BMI) and soft tissue thickness (ST thickness) (Kawchuk et al., 2011). Finally, the impact of incorrect marker placement on marker-based spinal curvature measurement was investigated.

## Materials and Methods

### Participants

Twenty patients with ASD were included from the local outpatient spinal clinic as well as 10 adults with normal spinal alignment (Table 1). Inclusion criteria for both groups were a minimum age of 18 years, whereas for the ASD group, participants had to present at least one of the following radiographically confirmed spinal deformity signs: pelvic tilt (PT) ≥20°, pelvic incidence minus lumbar lordosis (PI-LL) ≥10°, sagittal vertical axis (SVA) ≥4 cm, or coronal Cobb angle ≥20°. All subjects provided informed consent and the study protocol was approved by the local ethics committee (no. S58082).

**TABLE 1:** Subject characteristics, body morphology, and radiography.

	ASD (*n* = 20)	Control (*n* = 10)	*p*-value
**Subject characteristics**			
Age (year)	60.5 (13.5)	65.0 (8.3)	0.350
Gender (F/M)	14F/6M	7F/3M	1.000
**Body morphology**			
Height (cm)	163.8 (8.8)	167.5 (16.8)	0.719
Weight (kg)	66.5 (13.6)	63.7 (23.1)	0.510
BMI (kg/m^2^)	24.4 (5.1)	22.5 (5.4)	0.281
ST thickness (mm)	21.5 (12.8)	16.9 (9.8)	0.373
**Radiographic parameters**			
PT (°)	25.1 (12.4)	19.5 (9.9)	0.267
SVA (mm)	31.3 (35.0)	8.8 (13.5)	**0.005**
PI-LL (°)	9.7 (28.0)	−0.4 (14.0)	**0.029**
Coronal (D/T/L/N)	7D/11L/2N	10N	**<0.001**

*Medians and interquartile ranges are reported; Significance: p < 0.05. BMI, Body Mass Index; F, Female; M, Male; PT, Pelvic tilt; SVA, Sagittal vertical axis; PI, Pelvic incidence; LL, Lumbar lordosis; Coronal, SRS-Schwab Coronal classification; D, Double; T, Thoracic; L, Lumbar; N, No Major Coronal Deformity; ST, Soft tissue.*

### Data Collection Procedures

**FIGURE 1:**
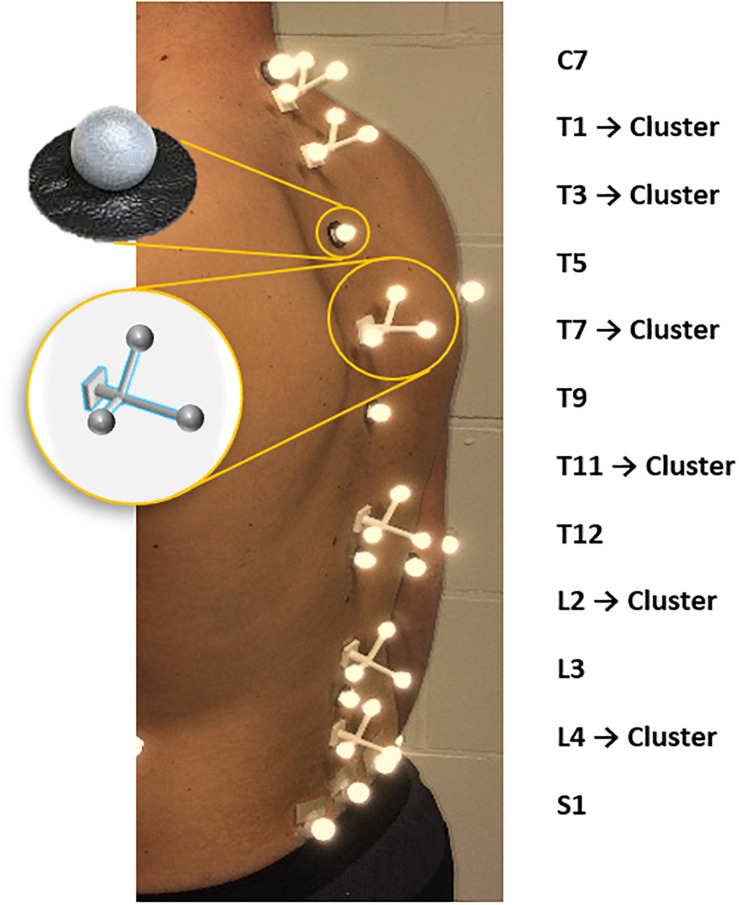
Spinal marker protocol.

A trained physiotherapist (5 years of experience in motion analysis) equipped all subjects, through manual palpation with the subject standing upright, with six single retro-reflective markers placed on the spinous processes of C7, T5, T9, T12, L3 and on the sacrum (in the middle between left and right posterior superior iliac spine) as well as six clusters, each consisting of three markers, placed on the spinous processes of T1, T3, T7, T11, L2, L4 (Overbergh et al., 2020; Severijns et al., 2020; Figure 1). All subjects underwent a full-spine biplanar radiographic examination (EOS imaging, Paris, France) in the finger-on-clavicle position. The subjects were positioned by an experienced staff member of our in-house radiology department, so that the subject coordinate system was as closely aligned as possible with the coordinate system of the EOS system. Subsequently, for all subjects a static motion capture trial was recorded in a standing position with the arms hanging alongside the body in the motion lab (Vicon, Oxford, United Kingdom).

**FIGURE 2:**
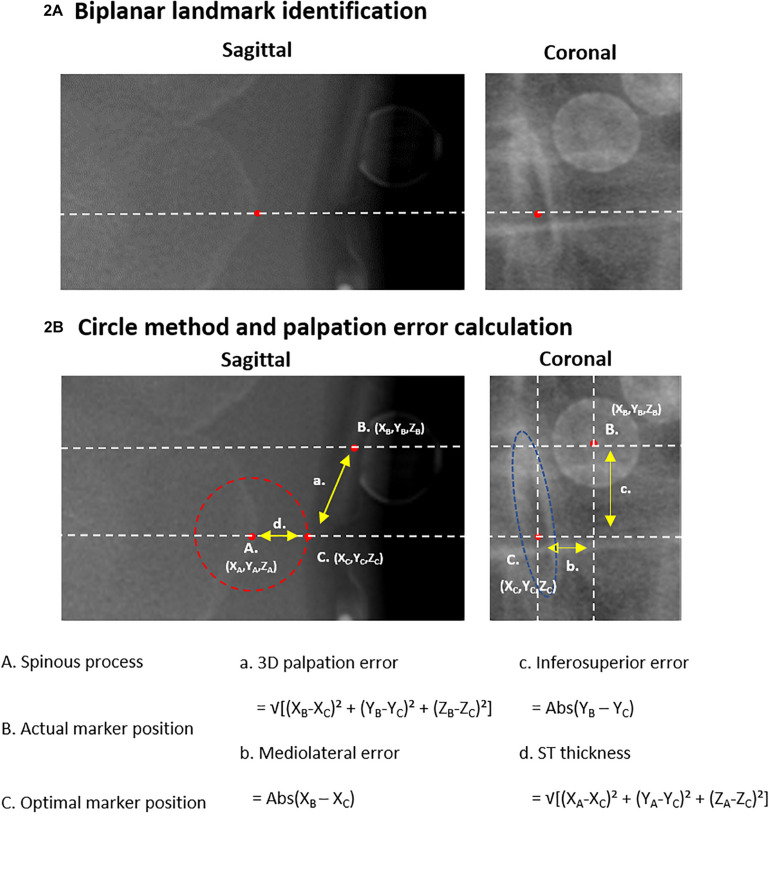
Palpation error. **(A)** The identification of a landmark in the sagittal image with corresponding reference line and landmark identification in the coronal image is displayed. **(B)** Shows the circle-based method to define the theoretical optimal marker position (‘C.’). The spinous process (‘A.’) serves as the center of the circle. The 3D distance between the actual marker position (‘B.’) and the optimal marker position (‘C.’) defines the palpation error. 3D, three-dimensional; X, mediolateral axis; Y, inferosuperior axis; Z, anteroposterior axis.

**FIGURE 3:**
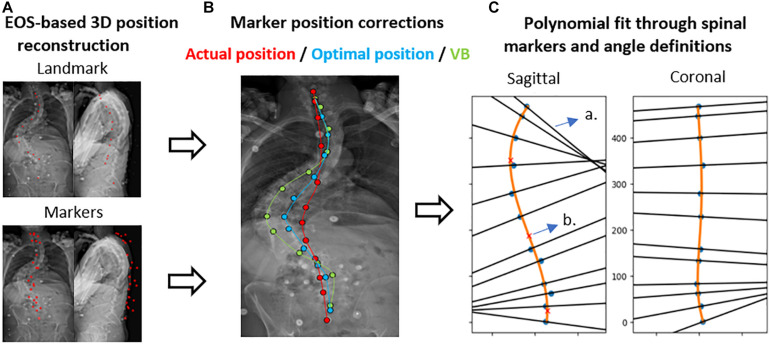
Polynomial method with subject-specific marker position correction. **(A)** Displays the EOS-based 3D reconstruction of markers and (anatomical) landmarks. **(B)** The marker position correction method toward the actual/optimal marker position or the vertebral body (VB) is presented. **(C)** Shows the polynomial fit and spinal angle definitions. a. Normal to the polynomial; b. Inflection point of the curve [figure edited from Severijns et al. (2020)].

The radiographic images were used to determine the sagittal spinopelvic alignment [PT, PI-LL, SVA and lumbar lordosis (LL)] as well as the type and severity of the coronal deformation according to the SRS-Schwab coronal classification (Schwab et al., 2012) and the method of Cobb (scoliosis) (Cobb, 1948), respectively. The images also served as data source for the assessment of spinal palpation error (see section “Palpation Error Quantification”; Figure 2), whereas the obtained motion capture-based marker trajectories were used to quantify the impact of marker misplacement on spinal alignment measurement (see section “Marker-Based Spinal Alignment Measurement and Impact of Incorrect Marker Placement”; Figure 3).

### Palpation Error Quantification

Palpation error was only evaluated for thoracolumbar (T11, T12) and lumbar (L2, L3, L4) levels instrumented with a marker, as thoracic spinous process identification on the sagittal radiographic images was restricted by superimposition of other structures, mainly the rib cage.

3D positions of both markers and anatomical landmarks were extracted from biplanar radiographic images. One single person, trained in analyzing radiographic images, manually identified the following three points from the sagittal and coronal radiographic images for each selected vertebral level (Overbergh et al., 2020; Figure 2):

A. Spinous process: The most posterior point of the spinous process identified on the sagittal image, as well as, on the same height, the midpoint of the spinous process identified on the coronal image.

B. Actual marker position: The midpoint of the base of the single marker (T12, L3) or marker cluster (T11, L2, L4) identified on both the sagittal and coronal images.

C. Optimal marker position: A theoretically optimal palpation would result in a marker placed as close as possible to the targeted anatomical landmark, i.e., the distance between the marker base and the landmark should be as small as possible to enable optimal tracking. We therefore defined the position on the skin at the closest distance from the spinous process point to the skin surface as the optimal marker position on the sagittal image. Thereto, a circle was centered on the spinous process point whereof the radius was enlarged until the circle edge reaches the skin surface (Figure 2B). The midpoint of the spinous process, in the same inferosuperior position as the sagittal defined position, was then defined as the corresponding optimal marker position in the coronal image.

Palpation error was defined as the 3D Euclidean distance between the actual (B.) and optimal (C.) marker positions, which further served as a basis for the calculation of mediolateral and inferosuperior palpation errors. All measurements were performed with respect to the EOS reference axis system. Palpation errors on each marker-instrumented level (T11, T12, L2, L3, L4), as well as the mean and maximum errors were reported. The 3D distance between the spinous process (A.) and the optimal marker position (C.) was used to quantify ST thickness. Maximal ST thickness was reported and used for further analysis (see section “Palpation Error Quantification and Correlations With Radiographic Parameters, BMI and ST Thickness”).

When the lower/upper boundary of the marker was above/below the upper/lower boundary of the spinous process, the palpation was identified as an incorrect level identification (Schmid et al., 2016). These were counted per level and the total percentage of incorrect level identifications per group (ASD vs. Control) was calculated.

### Marker-Based Spinal Alignment Measurement and Impact of Incorrect Marker Placement

Marker-based spinal alignment, namely LL and scoliosis, was measured using a previously validated method, in which a polynomial is fit through the marker positions of a motion trial (Severijns et al., 2020). The method allows for marker position correction using 3D coordinates of both the markers and anatomical landmarks derived from biplanar images (Figure 3). The polynomial order (2nd – 7th) was subject-specific and was identified through visual inspection of the best agreement with the corrected 3D marker positions (Supplementary Figure 1). More details on this method are reported elsewhere (Severijns et al., 2020). To assess the impact of palpation error on spinal alignment measurement, the agreement was investigated between LL and scoliosis measured by a polynomial fit through (1) the uncorrected marker positions, (2) the palpation error-corrected marker positions (toward the optimal marker position), and (3) the anatomically corrected marker positions (toward the vertebral body), respectively, and their radiographic equivalents (ground truth) as measured with the method of Cobb (1948).

### Statistical Analysis

Due to non-normality of a large part of the data (verified by the Shapiro–Wilk test), data were reported as medians and interquartile ranges and all statistical analyses were carried out using non-parametric methods. To compare the subject characteristics, radiographic parameters, palpation error and marker-based spinal alignment parameters between the ASD and control group, Mann–Whitney *U* tests were performed. To compare spinal alignment parameters between different methods within each group, Friedman tests were used. The relationship between marker-based and radiographic spinal alignment measurements were investigated using Spearman correlation coefficients (r_s_) and root mean square errors (RMSE). In addition, Spearman correlation coefficients were used to investigate the relationship between the mean palpation error and radiographic parameters (LL and scoliosis), BMI and ST thickness, respectively. Correlation coefficients of less than 0.25 were thereby considered as little to no relationship, from 0.25 to 0.50 as fair, from 0.50 to 0.75 as moderate to good and above 0.75 as good to excellent (Portney and Watkins, 2009). SPSS 26 (IBM Corp., Armonk, NY, United States) was used for statistical analysis. The level of significance was set at *p* < 0.05.

## Results

### Participants

The ASD and control groups did not differ in age, height, weight, BMI, and gender (Table 1). Radiographic parameters showed group differences for SVA (ASD: 31.3 mm; Control: 8.8 mm; *p* = 0.005), PI-LL (ASD: 9.7°; Control: −0.4°; *p* = 0.029), and coronal curve type (*p* < 0.001) but not for ST thickness.

### Palpation Error Quantification and Correlations With Radiographic Parameters, BMI and ST Thickness

The 3D palpation error showed no differences between the two groups on any spinal level, nor did the mean or maximum 3D palpation errors (Table 2). Comparing the mediolateral and inferosuperior errors separately, on the other hand, revealed larger mediolateral errors in the ASD group for the mean (ASD: 6.8 mm; Control: 2.5 mm; *p* = 0.002) and maximum (ASD: 12.6 mm; Control: 5.0 mm; *p* = 0.003) errors, and more specifically, for the spinal levels T12 (ASD: 8.7 mm; Control: 2.9 mm; *p* = 0.007) and L3 (ASD: 5.8 mm; Control: 2.3 mm; *p* = 0.015). Inferosuperior palpation errors were comparable between the two groups. The percentage of incorrect level identification was 37% for the ASD group, whereby mostly the lumbar levels L2 – L4 were incorrectly identified. In the control group, 32% of the markers were at least one level off, with an equal distribution across all spinal levels.

**TABLE 2:** 3D, mediolateral and inferosuperior palpation errors and incorrect level identifications.

	3D palpation error (mm)			Incorrect level identification		
Level	ASD (*n* = 20)	Control (*n* = 10)	*p*-value	ASD		Control
T11	8.9 (16.8)	12.5 (11.4)	0.307	3/20		4/10
T12	15.6 (15.0)	15.2 (11.9)	0.983	3/20		3/10
L2	21.7 (12.9)	13.8 (12.9)	0.055	12/20		4/10
L3	14.0 (12.3)	11.6 (10.0	0.248	10/20		3/10
L4	11.7 (13.6)	11.6 (11.4)	0.914	9/20		2/10
Mean PE	15.5 (9.2)	14.0 (5.8)	0.502	Total:		
Max PE	25.4 (12.0)	19.4 (12.2)	0.100	37%		32%

	**Mediolateral palpation error (mm)**			**Inferosuperior palpation error (mm)**		
**Level**	**ASD**	**Control**	***p*-value**	**ASD**	**Control**	***p*-value**

T11	4.6 (8.8)	3.2 (4.6)	0.155	5.0 (6.9)	10.7 (14.3)	0.074
T12	8.7 (16.2)	2.9 (2.4)	**0.007**	8.2 (11.4)	12.6 (12.4)	0.100
L2	8.5 (14.3)	3.1 (3.5)	0.143	15.7 (13.9)	11.4 (7.7)	0.846
L3	5.8 (8.5)	2.3 (3.5)	**0.015**	9.1 (16.9)	10.6 (8.9)	0.530
L4	3.7 (6.2)	2.1 (5.1)	0.422	8.1 (11.5)	9.5 (10.7)	0.502
Mean PE	6.8 (9.1)	2.5 (1.9)	**0.002**	8.1 (9.2)	12.4 (6.1)	0.091
Max PE	12.6 (17.4)	5.0 (4.6)	**0.003**	18.5 (12.0)	18.4 (12.9)	0.948

*Medians and interquartile ranges are reported. Significance: p < 0.05. 3D, Three-dimensional; PE, Palpation error; Mean, Mean palpation error over the different levels; Max, Maximal palpation error over the different levels.*

In the ASD group, the mediolateral palpation error showed a good to excellent relation with scoliosis (*r*_s_ = 0.77; *p* < 0.001) (Table 3). In the control group, the mediolateral palpation error showed a good to excellent relation with BMI (*r*_s_ = 0.78; *p* = 0.008) and a moderate to good relation with ST thickness (*r*_s_ = 0.66; *p* = 0.038).

**TABLE 3:** Correlations between mean palpation error and radiographic parameters/body morphology.

	3D palpation error				Incorrect level identifications			
	ASD		Control		ASD		Control	
	r_s_	*p*-value	r_s_	*p*-value	r_s_	*p*-value	r_s_	*p*-value
LL (°)	−0.17	0.466	0.22	0.533	−0.03	0.907	−0.21	0.566
Scoliosis (°)	0.19	0.416	N.A.		−0.24	0.313	N.A.	
BMI (kg/m^2^)	0.18	0.443	0.36	0.310	0.15	0.520	0.01	0.972
ST thickness	0.27	0.251	0.13	0.726	0.23	0.324	0.01	0.972

	**Mediolateral palpation error**				**Inferosuperior palpation error**			

LL (°)	0.34	0.141	−0.08	0.829	−0.22	0.359	0.12	0.751
Scoliosis (°)	0.77	**<0.001**	N.A.		−0.27	0.246	N.A.	
BMI (kg/m^2^)	−0.08	0.734	0.78	**0.008**	0.14	0.548	0.26	0.467
ST thickness	0.33	0.158	0.66	**0.038**	0.04	0.865	0.08	0.829

*Correlation coefficients are reported. Significance: p < 0.05. 3D, Three-dimensional; ASD, Adult Spinal Deformity; r_*s*_, Spearman correlation coefficient; BMI, Body Mass Index; LL, Lumbar Lordosis; ST, Soft tissue; N.A., Not applicable.*

### Impact of Marker Misplacement on Marker-Based Spinal Alignment Measurement

Due to problematic marker visibility on biplanar images, necessary for marker position correction, four subjects were excluded from the marker-based spinal alignment measurement. The following results are therefore based on a group of 16 patients with ASD and compared to ten control subjects.

Although all methods were able to discriminate ASD from controls on LL measurement, significant differences between methods were observed (Table 4). A polynomial through both the uncorrected and palpation error-corrected marker positions resulted in significantly lower LL and scoliosis values compared to radiographic values (*p* < 0.001), except for palpation error-corrected LL values in control subjects.

**TABLE 4:** Spinal alignment measurement with radiography and marker-based polynomial measurement, with different levels of marker position correction.

Parameter	ASD (*n* = 16)	Control (*n* = 10)	*p*-value between groups
**Lumbar lordosis (°)**			
1. Radiography	45.7 (38.9)	59.9 (13.0)	**0.027**
2. Polynomial method:			
a. No correction	22.7 (26.9)	32.9 (9.3)	**0.003**
b. Palpation error correction	26.4 (26.7)	38.6 (11.9)	**0.003**
c. Vertebral body correction	42.6 (38.7)	60.6 (16.6)	**0.017**
*p*-value between methods	**<0.001**	**<0.001**	
	(2a vs. 1 and 2c)	(2a vs. 1 and 2c)	
	(2b vs. 1 and 2c)	(2b vs. 2c)	
**Scoliosis (°)**			
1. Radiography	48.3 (29.7)	*N/A*	*N/A*
2. Polynomial method:			
a. No correction	7.4 (9.6)	*N/A*	*N/A*
b. Palpation error correction	16.5 (16.4)	*N/A*	*N/A*
c. Vertebral body correction	44.8 (36.9)	*N/A*	*N/A*
*p*-value between methods	**<0.001**		
	(2a vs. 1 and 2c)		
	(2b vs. 1)		

*Median (interquartile range); N/A, Not applicable; Significance: p < 0.05.*

For LL in the ASD group, a moderate correlation was found between corrected marker-based results and radiographic analysis (*r*_s_ = 0.71; *p* = 0.002), and a good correlation between uncorrected marker-based results and radiography (*r*_s_ = 0.76; *p* = 0.001) (Table 5). The RMSE was smaller for palpation error correction (RMSE = 21.48°) compared to no correction (RMSE = 27.18°). For scoliosis, correction for palpation error led to an excellent correlation with radiography (*r*_s_ = 0.83; *p* < 0.001) and a decreased RMSE (30.25°), compared to no correction (*r*_s_ = 0.50; *p* = 0.034; RMSE = 41.51°). For all parameters and in all groups, a polynomial through the vertebral body positions led to the highest correlation with radiography (LL_ASD_: *r*_s_ = 0.94; LL_control_: *r*_s_ = 0.90; Scoliosis: *r*_s_ = 0.92; *p* < 0.001), and the smallest RMSE (LL_ASD_: 7.21; LL_control_: 4.34; Scoliosis: 9.31).

**TABLE 5:** Relation between marker-based spinal alignment measurement and radiographic measurement.

Polynomial method:	Group	Correlation coefficient r_s_	*p*-value	RMSE
Lumbar lordosis (°)				
No correction	Control	0.32	0.365	27.11
	ASD	0.76	**0.001**	27.18
Palpation error correction	Control	0.33	0.347	23.03
	ASD	0.71	**0.002**	21.48
Vertebral body correction	Control	0.90	**<0.001**	4.34
	ASD	0.94	**<0.001**	7.21
Scoliosis (°)				
No correction	ASD	0.50	**0.034**	41.51
Palpation error correction	ASD	0.83	**<0.001**	30.25
Vertebral body correction	ASD	0.92	**<0.001**	9.31

*r_*s*_, Spearman correlation coefficient; RMSE, Root mean square error; Significance: p < 0.05.*

## Discussion

In this study, the 3D spinal palpation error and its impact on marker-based spinal alignment measurement were investigated and compared between patients with ASD and healthy controls. The results showed differences in palpation accuracy between deformed and healthy spines, with mean and maximum mediolateral errors of 6.8 mm and 12.6 mm in the ASD group and 2.5 mm and 5.0 mm in the control group, respectively. Furthermore, the mediolateral palpation error showed high correlations with scoliosis in the ASD group, and with BMI and ST thickness in the control group.

The high positive correlation between the mean mediolateral error and scoliosis indicates that the underlying cause of these errors can be assumed to be the deformity itself. Scoliosis is a 3D deformity, including a shift of the vertebral column in the coronal plane and a rotation in the transverse plane (Kim et al., 2010). Consequently, this rotation turns the spinous processes more toward the concave side of the curve, making their location less predictable compared to non-deformed spines. The largest mediolateral palpation errors were observed for spinal levels T12, L2, and L3, which corresponds to the levels where the apex of thoracolumbar/lumbar scoliosis curves is typically located (Lenke, 2007). Since the apex is the point of the curve with the largest coronal shift and the most vertebral rotation, this indeed explains the large mediolateral palpation errors for these spinal levels (Kim et al., 2010).

Surprisingly, in the inferosuperior direction no differences in palpation error were observed between deformed and healthy spines. This was also reflected by the percentages of incorrect level identifications, which were quite similar between both groups (ASD: 37%; Controls: 32%). With 32% in healthy spines, incorrect level identification was lower compared to Harlick et al. (2007), reporting 53% in the lumbar spine, but similar to Cooper et al. (2013), reporting a 29% incorrect palpation of L4. Schmid et al. (2015) reported 42.3% incorrect inferosuperior palpation across all spinal levels and 40% in lumbar levels in AIS, which corresponds to the palpation accuracy in our study in ASD. Comparing palpation errors between studies is challenging due to the heterogeneous methodologies applied in the literature. In this study, a very strict procedure was used, in which one point was identified as the optimal marker location and any deviation from this point was addressed as an error. Since this is, to the best of our knowledge, the first study to assess the 3D distance between actual and optimal spinal marker positions, preventing direct comparisons with the literature, mediolateral and inferosuperior 2D distances were also calculated. Other studies indeed used less strict methods, in which any overlap between the marker and the boundaries of the spinous process was identified as correct palpation (Harlick et al., 2007; Cooper et al., 2013; Schmid et al., 2015). This might explain the differences in mean mediolateral errors between the lumbar results of Schmid et al. (2015) in AIS (0.9 mm) and this study (6.8 mm). Moreover, AIS is characterized by deformities mostly affecting the thoracic spine (Konieczny et al., 2013) in contrast with more thoracolumbar and lumbar deformities in ASD (Acaroğlu et al., 2016), possibly also contributing to the larger lumbar palpation errors in this study.

Although palpation error was found to be mainly related to radiographic parameters in the patient group, mediolateral error in healthy spines instead showed higher correlations with BMI (*r* = 0.78) and ST thickness (*r* = 0.66). Such relation between BMI and ST thickness has been established previously (Kawchuk et al., 2011). Our results extrapolate and confirm the impact of higher BMI and larger ST thickness on palpation accuracy. Also in the ASD group, fair non-significant relations were found between palpation error and ST thickness. ST thickness is known to be increased in the lumbar spine compared to more proximal spinal levels, and changes depending on spinal position (Beaudette et al., 2017). As such, the combination of a lumbar deformity with increased lumbar ST thickness, might explain the relatively higher proportion of incorrect level identifications in the lumbar levels within the ASD group. Controls, having a neutrally positioned lumbar spine, had a more consistent ratio of incorrect level identification over thoracolumbar/lumbar levels. From a clinical perspective, these findings indicate that spinal palpation of lumbar levels for symptomatic level identification (Simmonds and Kumar, 1993) or intervertebral motion assessment (Nyberg and Russell Smith, 2013) is less accurate in deformed spines compared to non-deformed spines. The overall incorrect level identification results (ASD: 37%; Controls: 32%) also stress the importance of medical imaging guidance when identifying spinal levels for injections (Broadbent et al., 2000).

3D measurement of palpation error, allowed us to assess the impact of incorrect marker placement on marker-based spinal alignment measurement using a validated polynomial method with marker position correction (Severijns et al., 2020). As mentioned in the literature (Schmid et al., 2015; Severijns et al., 2020), skin marker-based curve measurement led to an underestimation of radiographic spinal alignment measurements. When correction of palpation error was performed, this underestimation decreased, resulting in higher LL and scoliosis values, and lower RMSEs. For scoliosis, palpation error correction also resulted in an excellent correlation with radiographic results. Although the results confirm that overall, correcting toward the vertebral body positions provides the most accurate results (Severijns et al., 2020), this study shows that incorrect marker placement impacts skin marker-based curve measurement, especially in the coronal plane, and should be considered in kinematic result interpretation when no marker position correction is or can be performed.

A first limitation of this study is that, except for T11 and T12, palpation error of thoracic levels was not investigated. The reason was the superimposition of other structures on radiographic images, mainly the ribcage, preventing a reliable identification of the thoracic spinous processes. Consequently, these levels were not corrected for palpation error in the marker-based spinal alignment measurement. However, since ASD is mainly characterized by thoracolumbar and lumbar deformity (Acaroğlu et al., 2016), the clinically most relevant spinal levels were included in this study. A second limitation is the difference in subject positioning during marker placement (arms alongside the body) and biplanar imaging (fingers on the clavicles), resulting in slight differences in lumbar position (Marks et al., 2009). Consequently, skin motion artifact (Mahallati et al., 2016) cannot be excluded of having led to small differences in marker location during imaging. Indeed, our study design did not allow investigating the effects of skin motion artifacts on marker-based spinal alignment measurement during motion. Future research assessing these artifacts during different positions (semi-static) (Overbergh et al., 2020) or during a range of clinically relevant dynamic movements is required, to further increase confidence in marker-based spinal kinematic results during motion.

In conclusion, this study showed that, although 3D palpation error was similar between deformed and healthy adult spines, mediolateral palpation was less accurate in the ASD group. Overall accuracy of spinal level identification was similar across groups, however, with a larger inaccuracy in lumbar levels within the ASD group. Determining factors for palpation error were spinal malalignment, in particular scoliotic deformity, in deformed spines and body morphology (i.e., increased BMI and ST thickness) in healthy spines. Improved spinal alignment measurements after palpation error correction, shows the need for radiograph-based marker correction methods, and therefore should be considered when interpreting kinematic results.

## Data Availability Statement

The raw data supporting the conclusions of this article will be made available by the authors, without undue reservation.

## Ethics Statement

The studies involving human participants were reviewed and approved by the Commissie Medische Ethiek UZ KU Leuven/Onderzoek. The patients/participants provided their written informed consent to participate in this study.

## Author Contributions

PS collected the data, conceptualized the study, analyzed the data, and wrote the initial manuscript. TO collected the data, conceptualized the study, developed the technical tools necessary for data analysis, and edited the manuscript. SS conceptualized the study and edited the manuscript. LM conceptualized the study and was responsible for subject recruitment. LS conceptualized the study, edited the manuscript, and supervised the project. All authors contributed to the article and approved the submitted version.

## Conflict of Interest

The authors declare that the research was conducted in the absence of any commercial or financial relationships that could be construed as a potential conflict of interest.
