# Supplementary material for: Spinal Palpation Error and Its Impact on Skin Marker-Based Spinal Alignment Measurement in Adult Spinal Deformity
Source: Front Bioeng Biotechnol. 2021 Jun 23;9:687323. doi: 10.3389/fbioe.2021.687323 (PMC8281975; doi:10.3389/fbioe.2021.687323)
Supplement: Supplementary file 1 [file Data_Sheet_1.docx]

Supplementary Material

# Supplementary Figure


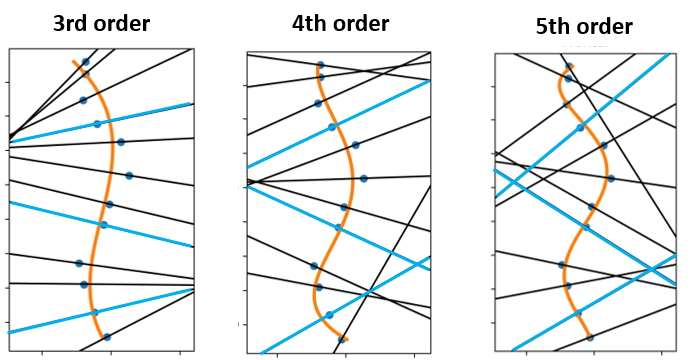


**Figure A1. Polynomial order identification.**

In this figure, three different orders (3rd – 5th) haven been fitted on the same marker position data. A visual inspection reveals that the 5th order polynomial provides the best fit. The blue lines show the levels of interest to measure scoliosis using the method of Cobb.
